# Modifiable and fixed factors predicting quality of life in people with colorectal cancer

**DOI:** 10.1038/bjc.2011.155

**Published:** 2011-05-10

**Authors:** N M Gray, S J Hall, S Browne, U Macleod, E Mitchell, A J Lee, M Johnston, S Wyke, L Samuel, D Weller, N C Campbell

**Affiliations:** 1 Centre of Academic Primary Care, University of Aberdeen, Foresterhill Health Centre, Westburn Road, Aberdeen AB25 2AY, UK; 2 General Practice & Primary Care, University of Glasgow, 1 Horselethill Road, Glasgow G12 9LX, UK; 3 Hull York Medical School, University of Hull, Hertford Building, Hull HU6 7RX, UK; 4 Aberdeen Health Psychology Group, University of Aberdeen, 2nd floor, Health Sciences Building, Aberdeen AB25 2ZD, UK; 5 Department of Nursing & Midwifery, University of Stirling, RG Bomont Building, Stirling FK9 4LA, UK; 6 Anchor Unit, Aberdeen Royal Infirmary, Foresterhill Road, Aberdeen AB25 2ZP, UK; 7 Centre for Population Health Sciences, University of Edinburgh, Room 309, Doorway 1, Medical Quad, Teviot Place, Edinburgh EH8 9AG, UK

**Keywords:** quality of life, colorectal cancer, assessment

## Abstract

**Background:**

People with colorectal cancer have impaired quality of life (QoL). We investigated what factors were most highly associated with it.

**Methods:**

Four hundred and ninety-six people with colorectal cancer completed questionnaires about QoL, functioning, symptoms, co-morbidity, cognitions and personal and social factors. Disease, treatment and co-morbidity data were abstracted from case notes. Multiple linear regression identified modifiable and unmodifiable factors independently predictive of global quality of life (EORTC-QLQ-C30).

**Results:**

Of unmodifiable factors, female sex (*P*<0.001), more self-reported co-morbidities (*P*=0.006) and metastases at diagnosis (*P*=0.036) significantly predicted poorer QoL, but explained little of the variability in the model (*R*^2^=0.064). Adding modifiable factors, poorer role (*P*<0.001) and social functioning (*P*=0.003), fatigue (*P*=0.001), dyspnoea (*P*=0.001), anorexia (*P*<0.001), depression (*P*<0.001) and worse perceived consequences (*P*=0.013) improved the model fit considerably (*R*^2^=0.574). Omitting functioning subscales resulted in recent diagnosis (*P*=0.002), lower perceived personal control (*P*=0.020) and travel difficulties (*P*<0.001) becoming significant predictors.

**Conclusion:**

Most factors affecting QoL are modifiable, especially symptoms (fatigue, anorexia, dyspnoea) and depression. Beliefs about illness are also important. Unmodifiable factors, including metastatic (or unstaged) disease at diagnosis, have less impact. There appears to be potential for interventions to improve QoL in patients with colorectal cancer.

Colorectal cancer is the third most common cancer in the UK and, with advances in treatment, more people are not only being cured, but also surviving for longer with the disease (http://info.cancerresearchuk.org/cancerstats/incidence/commoncancers/). Treatment almost always involves surgery, can result in stomas and is frequently accompanied by chemotherapy, radiotherapy or both (SIGN, 2003). The adverse effects of both the disease and its treatment can be longstanding, including lack of energy, bowel problems, poor body image and emotional problems (Phipps *et al*, 2008), as well as sleep difficulties, fear of recurrence, anxiety, depression, sensory neuropathy, gastrointestinal problems, urinary incontinence and sexual dysfunction (Denlinger and Barsevick, 2009). Moreover, most people with colorectal cancer are older and may have functional limitations, geriatric syndromes and other significant conditions (including heart disease, chronic obstructive pulmonary disease and other cancers) that are likely to affect how they feel (Koroukian *et al*, 2010). Improving their quality of life (QoL) will, then, be complex, having to take account of multiple factors related to the cancer, its treatment, other conditions, social factors and the person's response. To tackle this challenge, we need first to identify the potentially modifiable and fixed factors most associated with better or worse QoL.

Previous research has helped to highlight some factors associated with poorer QoL in colorectal cancer (Steginga *et al*, 2009). People with more advanced disease (Sharma *et al*, 2007), women (Pucciarelli *et al*, 2008) and those with certain symptoms, especially diarrhoea and constipation (Wilson *et al*, 2006), report poorer QoL. However, much evidence is conflicting or inconclusive – for example, stomas may or may not be detrimental to QoL (Wilson *et al*, 2006; Cornish *et al*, 2007; Ross *et al*, 2007; Wilson and Alexander, 2008; Bloemen *et al*, 2009; Krouse *et al*, 2009; Yau *et al*, 2009), older people may have better or worse QoL than younger people (Pucciarelli *et al*, 2008; Wilson and Alexander, 2008) and there are suggestions that non-disease factors may be more important than disease factors (Siassi *et al*, 2009).

This study was conducted to inform attempts to improve the QoL of people with colorectal cancer. It was undertaken as part of a programme of work to establish the potential for primary care to tackle social inequalities for people with colorectal cancer. The aims were to (1) quantify the health-related quality of life (HRQOL) in people with a confirmed diagnosis of colorectal cancer and (2) identify both modifiable and unmodifiable (or fixed) factors associated with poorer HRQOL.

## Materials and methods

### Participants

Participants recently diagnosed with colorectal cancer were recruited in North East Scotland, via the colorectal cancer multidisciplinary team, and in Glasgow, via the colorectal specialist nurses. In addition, participants 1–2 years into follow up were recruited in North East Scotland via colorectal oncology and surgical outpatient clinics. Participants were eligible for inclusion in the study if they had had a definitive diagnosis of colorectal cancer, and had commenced their initial treatment (normally surgery or, in non-resectable cases, palliative radiotherapy or chemotherapy). Participants were excluded from the study if they were unable to give informed consent or complete the questionnaire (e.g., due to dementia), or, in the opinion of their clinical team, had a life expectancy of <1 month.

Eligible participants were initially approached by a member of the clinical team treating them. Willing participants were then contacted by the researchers who sought written informed consent for participation. Participants were asked to complete a questionnaire booklet (see below). Most questionnaires were completed during face-to-face interviews with one of the research team. However, some participants, who stated a preference or lived long distances away (e.g., in Orkney or Shetland), self-completed the questionnaires or were interviewed by telephone.

This project was reviewed and fully approved by the Multi-Centre Research Ethics Committee for Scotland, Committee A.

### Materials

The main outcome variable was global HRQOL, measured using the EORTC-QLQ-C30 (http://www.eortc.be/home/qol/). This is a validated and widely used instrument to measure overall HRQOL in people with cancer. The QLQ-C30 comprises five functional scales (physical, role, cognitive, emotional and social), three symptom scales (fatigue, pain and nausea and vomiting), and a number of single items assessing additional symptoms commonly reported by people with cancer (dyspnoea, loss of appetite, insomnia, constipation and diarrhoea) and perceived financial impact of the disease. These additional scales/items were included in the analysis as independent variables.

Other independent variables for inclusion in the analysis were collected by questionnaire and abstraction from case notes. The Hospital Anxiety and Depression scale (HADS) (Zigmond and Snaith, 1983) was used to measure participant levels of anxiety and depression. The revised Illness Perception Questionnaire (IPQ-R) was used to assess six components of illness representation in Leventhal's common sense self-regulation model, which has been previously found to predict coping, health seeking behaviour, adherence to treatment, mood and functional adaptation (Moss-Morris *et al*, 2002; Hagger and Orbell, 2003). The Social Difficulties Inventory (SDI) was used to identify individuals with social problems (Wright *et al*, 2007).

The personal and social data collected included date of birth, postcode, living arrangements (i.e., living on own, living with spouse, living with others), dependents, ethnicity, education, employment status, home ownership and annual income. A small area-based measure of deprivation (Carstairs quintile grouping) was assigned to participants according to postcode of residence. The urban/rural status of the participant's postcode was allocated using the 2005–2006 Scottish Executive Urban Rural 6-category Classification (http://www.scotland.gov.uk/Publications/2006/07/31114822/UR2006downloads), participants were defined as living in an accessible or remote area. Participants’ travelling time from home to nearest cancer centre was calculated using a cutoff of 60 min travelling time to categorise participants.

Participants were also asked about their health and any illnesses they may have had. This included questions related to co-morbidities, smoking habits and current pain/discomfort. Participant-reported (PR) co-morbidities were included in the analyses firstly, as a count of the number of co-morbidities, and secondly, for more common diseases (with prevalence ⩾10%) as an individual co-morbidity. To complement information from questionnaires, data were also collected from general practice (GP) and hospital case notes on disease stage, treatments undertaken and co-morbidities.

Time from date of diagnosis to completion of the questionnaire was used to categorise participants into either (1) ‘newly diagnosed’ (questionnaire completed up to 26 weeks from date of diagnosis) or (2) ‘follow up’ (questionnaire completed 48 weeks or more from date of diagnosis). Fifteen participants falling outside these time frames were ineligible for the study and were coded as missing.

### Statistical methods

Descriptive statistics and histograms were produced for each of the outcome measures. The distribution of each scale was checked for normality and descriptive statistics were examined to check for skewness. The HADS scores were categorised into case or non-case using a cutoff score of 8 or more to determine caseness for each of anxiety and depression (Bjelland *et al*, 2002). In order to facilitate interpretation, the subscale scores of the QLQ-C30 and the IPQ-R were split into tertiles. An SDI summary score was calculated using 16 items from the scale and dichotomised using a score below 10 to indicate ‘no difficulties’ and a score of 10 or more to indicate ‘some difficulties’. The remaining five SDI scale items were dichotomised into ‘no difficulties’ and ‘some difficulties’.

The independent samples *t*-test and analysis of variance were used to test whether there were significant differences in the mean QoL scores across five groups of variables: (1) personal and social factors, (2) disease and treatment factors, (3) PR co-morbidities, (4) hospital and GP case note reported co-morbidities and (5) questionnaire responses, including QLQ-C30 function and symptom scales, social difficulties, illness perceptions and anxiety and depression. Variables with a *P*-value of 0.1 or less were included in multiple linear regression models to identify (for each of the five groupings) which factors were predictive of QoL score. Variables with a *P*-value of 0.1 or less from each of the multi-adjusted models were then included in a final multiple linear backward stepwise regression model to identify which factors remained independently predictive of QoL score. The final model was rerun on the full data set, entering first fixed variables and then modifiable ones to provide data on how much variability was explained by each.

The functioning subscales of the QLQ-C30 include questions about activity limitations and participation restrictions. These factors may to some extent mediate the effects of other factors on QoL, so the regression modelling was repeated omitting these scales to determine what effect that would have (if any) on the predictors of QoL score.

Throughout all statistical analyses, a two-sided *P*-value ⩽0.05 was used as the threshold for statistical significance.

## Results

A total of 496 participants are included in the analysis, 187 from Glasgow and 309 from North East Scotland (including the islands of Orkney and Shetland). One person was excluded due to incomplete questionnaire data. The mean age of participants was 66 (s.d., 11.11 years); over 70% of participants were over the age of 60. Descriptive statistics for all of the variables considered in the analyses are provided in Supplementary Tables 1–5.

**Figure 1:**
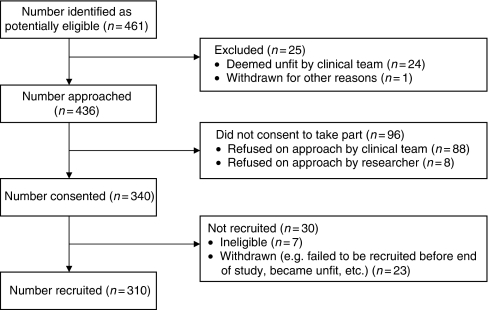
Flowchart of numbers of participants approached, consented and recruited to the study in North East Scotland.

In Glasgow, participants were identified by five clinical staff members in three hospitals; clinical staff did not retain details of who had been approached; thus, it was not possible to calculate recruitment or consenting rates. In Grampian, recruitment was from four hospitals; eligible participants were identified by researchers and then approached by a member of the clinical team. Of 436 eligible patients, 310 (71%) were recruited (Figure 1). The mean QoL score was 65.791 (s.d., 21.81).

### Personal and social factors (*R*^2^=0.068)

When the personal and social factors with a *P*-value of 0.1 or less in univariate analyses were modelled together sex, income, urban rural status and home ownership remained independently predictive of QoL score (Table 1). Thus, those with the lowest QoL scores were women, those who had lower incomes, lived in accessible areas and did not own their own home.

### Disease and treatment factors (*R*^2^=0.090)

Disease stage was categorised initially as (1) complete response to neoadjuvant chemo-radiotherapy before pathological staging (*n*=13), (2) Dukes A (*n*=67), (3) Dukes B (*n*=171), (4) Dukes C (*n*=151), (5) metastatic (*n*=71) or (6) unstaged (*n*=23). Initial univariate analyses showed few differences in QoL among participants with a complete response and those diagnosed with Dukes A through C. Participants with metastatic or unstaged disease at diagnosis had the poorest QoL. For the remaining analyses, staging was collapsed into two categories: (1) complete response through to Dukes C and (2) metastatic and unstaged. Shorter time since diagnosis was significantly associated with poorer QoL, but recurrence was not. Of the treatments received, surgery and palliative chemotherapy were both predictive of QoL univariately. Participants who had not received surgery (or for whom these data were missing) had the poorest QoL. Having a stoma was predictive of poorer QoL. In the final disease and treatment model, shorter time since diagnosis, metastatic (or unstaged) disease at diagnosis, no surgical treatment and presence of a stoma remained significantly predictive of a poorer QoL score (Table 1).

### PR co-morbidities (*R*^2^=0.027)

Participants who reported having more than one co-morbidity had the poorest QoL (Table 1). Of co-morbidities reported by 10% or more of the sample, heart disease, anxiety/depression and having another cancer (most often breast or prostate) were all significantly associated with lower QoL scores in univariate analyses. When modelled together, the number of PR co-morbidities and reporting having had another cancer were significant predictors of QoL score. Participants who reported having current pain also had poorer QoL.

### Case note recorded co-morbidities (*R*^2^=0.009)

These analyses were repeated for hospital and GP case note reported co-morbidities (Table 1). None of the hospital and GP case note reported co-morbidities were significantly associated with QoL score, nor was the number of co-morbidities recorded. The mental health and cancer variables were included in the final case note model because 10% or more of the sample had the disease and the *P*-value in univariate analyses was below 0.10. Having had another cancer remained in this model (*P*=0.067), and was taken forward into the final regression model.

### Function and symptoms (*R*^2^=0.537)

All of the QLQ-C30 functioning subscales were predictive of QoL score, as were the symptoms of fatigue, dyspnoea, appetite loss and diarrhoea (Table 2).

### Social difficulties (*R*^2^=0.183)

Participants who had experienced some social difficulties (SDI summary score) had lower QoL scores, as did those participants who had sexual difficulties, difficulty with where they lived or struggled with travel plans.

### Cognitions (illness perceptions) (*R*^2^=0.198)

The IPQ-R illness identity, timeline cyclical and consequences subscales were negatively predictive of QoL score. Thus, participants who attributed a high number of symptoms to their illness (illness identity), who considered their illness to be cyclical and who considered their illness to have negative consequences all had poorer QoL scores. Participants who scored highly on the personal and treatment control subscales had positive beliefs about the controllability of the illness and their QoL scores were significantly higher.

### Anxiety and depression (*R*^2^=0.271)

Both the anxiety and depression subscales of the HADS were significantly predictive of QoL score.

### Final model (*R*^2^=0.574)

When variables from all five groupings were modelled together, sex, number of self-reported co-morbidities, stage at diagnosis, role and social functioning, fatigue, dyspnoea, appetite loss, HADS depression and IPQ-R consequences were all significantly predictive of QoL score (Table 3). The fixed variables (sex, co-morbidities and disease stage) accounted for 6.4% of the variability in QoL score, with the remaining, modifiable variables accounting for a further 51%.

When the QLQ-C30 functioning subscales were removed from the model, time since diagnosis, SDI question 20 (relating to travel plans) and the IPQ-R personal control subscale entered the model and became significant predictors of QoL (*R*^2^=0.538) (Table 4).

## Discussion

We found that physical, psychological and social factors were all significantly and independently associated with overall QoL. Most predictors were modifiable, with symptoms, depression and limitations to usual activities being most important. The only important disease factor was having metastatic or unstaged disease at diagnosis. The difference in QoL between those with and without metastatic disease was around that regarded as clinically important; differences were larger for nearly all the statistically significant modifiable factors we identified. Women had poorer QoL and participants’ beliefs had a role.

**Figure 2:**
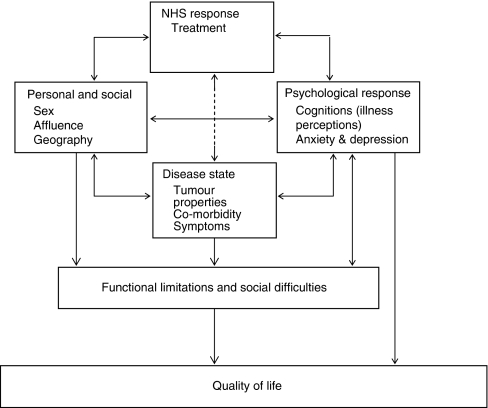
Model of biological, psychological and social factors contributing to QoL in colorectal cancer.

The study benefited from having as near as possible to a consecutive sample of participants. Its age and sex distribution reasonably approximated that of all people diagnosed with colorectal cancer in Scotland in 2007 (http://www.isdscotland.org/cancer). Our sample included a slightly greater proportion of younger participants compared with that for the whole of Scotland (9% *vs* 5% aged under 50 years) and slightly fewer participants aged 80 years or more (11% *vs* 25%). This may reflect our recruitment methods (via outpatient surgical and oncology clinics) and exclusion of people whose life expectancy was <1 month or who were unable to give informed consent (e.g., due to dementia). We acknowledge that our findings may not fully represent those people who were severely ill, elderly or who refused to take part. Our questionnaire data are self-report, but we were also able to include data from both hospital and GP case notes. Our data were cross-sectional and, thus, cannot reflect the variation in QoL that will inevitably occur over the cancer pathway. However, we did collect data from patients at different stages along the pathway, from very early diagnosis to 2 years post-diagnosis, in an attempt to capture the differences that might occur. The large data set we collected, including physical, psychological and social factors, has enabled us to construct a comprehensive picture of factors involved in QoL. A disadvantage of having so many variables is that they may occasionally be found to be significant by chance. However, when developing our programme of work, we attempted to map the biological, psychological and social mechanisms by which the QoL of people with colorectal cancer might be affected. *A priori* we identified the interactions among personal and social, NHS response, disease state and emotional response factors as being important determinants of QoL and this conceptual model was the driver for our approach to our analysis. Our work has further clarified the way in which these factors impact on an individual's QoL, as illustrated in Figure 2. It appears that the impact of personal and social, NHS response (treatment) and disease state factors on QoL are mediated by their impact on a person's emotional, physical and social functioning. This is consistent with the World Health Organisation's International Classification of Functioning, Disability and Health (WHO ICF) (http://www.who.int/classifications/icf/en/), which emphasises the importance of the impact of the disease.

Our findings on the effects of sex, stage of disease, symptoms, beliefs about consequences and co-morbidities are in line with a large previous Australian study despite different instruments and settings (Steginga *et al*, 2009). We used the symptom and functional domains of the EORTC-QLQ-C30 as predictor variables (rather than outcomes) to explore how ‘body function and structure’ and ‘activity and participation’, as classified by the WHO ICF, impacted on QoL. Thus, we confirmed that QoL is poorer soon after diagnosis compared with later (Arndt *et al*, 2006; Tsunoda *et al*, 2007; Wilson and Alexander, 2008) but, by exploring further, found that this difference was accounted for by activity limitations and participation restrictions (e.g., work, daily activities and hobbies, interference with family and social life) as measured by EORTC-QLQ-C30 functioning scales. These factors also partly (but not completely) mediated the effects of symptoms on QoL. The importance of symptoms has been reported before, but previous studies have focussed on diarrhoea, faecal control and constipation (Wilson *et al*, 2006; Steginga *et al*, 2009). We found that the two most important symptoms for QoL were fatigue and loss of appetite. In line with others (Tsunoda *et al*, 2007), we found that QoL was poorer for those with markers of deprivation, including lower income and no home ownership. That this association was not significant in our final model suggests it is mediated by symptoms, limitations to function, beliefs about the illness and co-morbidities. Previous studies have also found co-morbidities to be associated with poorer QoL, but have used either self-reported (Steginga *et al*, 2009) or medically recorded (Tsunoda *et al*, 2007) data. By exploring both, we found that self-reported co-morbidities are more important for QoL – especially heart disease, anxiety/depression and having had another cancer. We were unable to analyse less common co-morbidities individually, but the total number of co-morbidities reported was the most significant factor. We know that not everyone who experiences symptoms of a particular disorder (e.g., depression) seeks out medical care, so these may be missing in case notes. Conversely, self-report may ignore diseases that, while medically recorded, are not impacting on, or currently active for, the person. This finding may then reflect the tendency of people to focus on negative aspects, including those most affecting their QoL.

If we wish to improve QoL in people with colorectal cancer, then we need first to identify those most at risk, and second to intervene to address factors which are modifiable (Campbell *et al*, 2007). Our findings show that women, and those with metastatic (or unstaged) disease and/or multiple co-morbidities, are affected disproportionately and that QoL is worst in the first few months after diagnosis and initial treatment. The variability in QoL explained by these unmodifiable factors is, however, small and the remaining independent predictors appear to have potential for intervention. Symptoms and depression have strong impacts on QoL and these can be treated. We found fatigue to be common and particularly important; fatigue has responded in a variety of diseases, including cancer, to programmes of graded activity (Knols *et al*, 2005; Courneya and Friedenreich, 2007). Depression and anxiety in people with cancer have improved with nurse-led interventions, exercise and antidepressants (Strong *et al*, 2008). It is also possible, with appropriate theoretically grounded interventions, to tackle peoples’ beliefs, improving confidence and reducing negative thoughts about consequences (Johnston *et al*, 2007). Difficulties with travelling may be helped by interventions to reduce the need for it, such as more locally based follow up. Furthermore, if the effects of the above factors are at least partly mediated by limitations to usual activities (work, hobbies, etc.) and restrictions of participation in social and family life, then it may be possible to intervene at these levels to increase activities and participation even in the presence of the underlying factors. There is reason, then, to be positive about the potential for interventions to improve QoL in people with colorectal cancer.

Our findings add to a growing literature on QoL in colorectal cancer and show that there may be opportunities to improve it. Our challenge now is to develop interventions, which tackle symptoms and depression, reduce restrictions to activity and enable people to return to as full participation as possible. While these interventions will need rigorous evaluation, there is every reason to be optimistic for their future.

## Figures and Tables

**Table 1:** Associations between QLQ-C30 global quality of life score and personal and social factors, disease and treatment factors, PR co-morbidities and hospital and GP case note reported co-morbidities

	**Univariate analysis**			**Multivariate analysis**		
	**B**	**s.e.**	***P*-value**	**B**	**s.e.**	***P*-value**
*Personal and social factors (multi-adjusted* R^*2*^*=0.068)*^a^						
Sex	−7.180	1.945	<0.001	−5.255	2.197	0.017
Income	3.209	0.783	<0.001	2.201	0.838	0.009
Carstairs quintiles	−1.704	0.658	0.010			
Urban rural status	4.111	2.180	0.060	4.249	2.330	0.069
Home ownership	−9.186	2.388	<0.001	−6.719	2.860	0.019
						
*Disease and treatment factors (multi-adjusted* R^*2*^*=0.090)*^b^						
Time since diagnosis	11.118	2.088	<0.001	10.131	2.061	<0.001
Stage at diagnosis	−9.206	2.467	<0.001	−6.169	2.701	0.023
Surgery (none)	−10.290	3.453	0.003	−8.859	3.822	0.021
Palliative Chemotherapy	−5.760	2.953	0.052			
Stoma	−5.333	2.216	0.016	−4.720	2.231	0.035
						
*PR co-morbidities (multi-adjusted* R^*2*^*=0.027)*^c^						
Number of PR co-morbidities	−2.446	0.776	0.002	−1.890	0.807	0.020
Heart disease	−5.391	2.578	0.037			
Anxiety/depression	−6.345	3.109	0.042			
Cancer (other than bowel)	−8.636	2.724	0.002	−6.702	2.834	0.018
						
*Current pain*						
Current pain	−12.469	1.943	<0.001			
						
*Hospital and GP case note reported co-morbidities (multi-adjusted* R^*2*^*=0.009)*^d^						
Mental health	−4.003	2.410	0.097			
Cancer	−5.620	3.063	0.067	−5.620	3.063	0.067

Abbreviations: GP=general practice; PR=participant reported; QLQ=quality of life questionnaire.

a Factors not significant univariately (*P*>0.01): age, centre, education, travelling time, employment status and smoking status.

b Factors not significant univariately (*P*>0.01): site of cancer, recurrence, first contact (emergency versus non-emergency), neo-adjuvant chemotherapy, adjuvant chemotherapy, neo-adjuvant radiotherapy, adjuvant radiotherapy and palliative radiotherapy.

c Factors not significant univariately (*P*>0.01): heart disease, high blood pressure, diabetes and osteoarthritis.

d Factors not significant univariately (*P*>0.01): musculoskeletal, circulatory system, respiratory, gastrointestinal and endocrine.

**Table 2:** Associations between QLQ-C30 global quality of life score and the QLQ-C30 subscales, SDI, IPQ-R and HADS

	**Univariate analysis**			**Multivariate analysis**		
	**B**	**s.e.**	***P*-value**	**B**	**s.e.**	***P*-value**
*QLQ-C30 (adjusted* R^*2*^*=0.537)*						
Physical Function	−13.906	1.024	<0.001	−3.400	1.147	0.003
Role function	−16.224	0.992	<0.001	−6.586	1.225	<0.001
Emotional function	−9.608	1.096	<0.001	−3.141	0.912	0.001
Cognitive function	−10.917	1.133	<0.001	−2.170	1.009	0.032
Social function	−14.447	1.090	<0.001	−3.996	1.103	<0.001
Fatigue	−24.558	2.189	<0.001	−4.421	1.995	0.027
Nausea/vomiting	−20.030	2.064	<0.001	—	—	—
Pain	−16.131	1.823	<0.001	—	—	—
Dyspnoea	−16.928	1.940	<0.001	−3.646	1.650	0.028
Insomnia	−13.073	1.877	<0.001	—	—	—
Appetite loss	−23.089	1.882	<0.001	−10.920	1.631	<0.001
Constipation	−6.019	2.232	0.007	—	—	—
Diarrhoea	−9.753	1.997	<0.001	−2.835	1.441	0.050
Financial problems	−9.392	2.268	<0.001	—	—	—
						
*SDI (adjusted* R^*2*^*=0.183)*^a^						
Q13 difficulty with sexual matters	−7.328	2.153	0.001			
Q18 difficulty with where you live	−14.233	3.542	<0.001	−7.532	3.592	0.037
Q20 difficulty with travel plans	−13.098	1.884	<0.001	−9.742	1.876	<0.001
Q21 difficulty with any other area of everyday life	−13.388	2.298	<0.001			
SDI summary score (dichotomous)	−19.306	2.261	<0.001	−15.424	2.341	<0.001
						
*IPQ-R (tertiles) (adjusted* R^*2*^*=0.198)*						
IPQ illness identity	−7.000	1.120	<0.001	−4.029	1.131	<0.001
IPQ timeline	−3.155	1.108	0.005	—	—	—
IPQ timeline cyclical	−8.767	1.405	<0.001	−4.760	1.408	0.001
IPQ consequences	−9.208	1.068	<0.001	−6.444	1.135	<0.001
IPQ personal control	2.869	1.213	0.018	2.297	1.178	0.052
IPQ treatment control	5.475	1.149	<0.001	2.781	1.151	0.016
IPQ illness coherence	3.257	1.094	0.003	—	—	—
IPQ emotional representations	−8.364	1.485	<0.001	—	—	—
						
*HADS (adjusted* R^*2*^*=0.271)*						
Anxiety	−12.516	1.523	<0.001	−9.779	2.314	<0.001
Depression	−18.474	1.449	<0.001	−24.959	2.462	<0.001

Abbreviations: HADS=hospital anxiety and depression scale; IPQ-R=illness perception questionnaire; QLQ=quality of life questionnaire; SDI=social difficulties inventory.

a SDI factors not significant univariately (*P*>0.01): plans to have a family.

**Table 3:** Multivariate analysis of all factors from personal and social, disease and treatment, participant-reported co-morbidities, hospital and GP case note reported co-morbidities models and questionnaire models

	**Including QLQ-C30 functioning scales (multi-adjusted – *R*^2^=0.574)**				
	**Standardised coefficients *β***	**B**	**s.e.**	***P*-value**	**Cumulative *R*^2^**
*Fixed variables*					
Metastatic (or unstaged) disease at diagnosis	−0.065	−3.574	1.703	0.036	0.024
Number of self-reported co-morbidities	−0.085	−1.483	0.539	0.006	0.044
Sex	−0.119	−5.214	1.331	<0.001	0.064
					
*Modifiable variables*					
Role function	−0.267	−7.255	1.078	<0.001	0.399
HADS depression	−−0.215	−12.460	1.977	<0.001	0.477
Appetite loss	−0.192	−9.162	1.589	<0.001	0.530
Fatigue	−0.115	−6.214	1.897	0.001	0.548
Social function	−0.120	−3.352	1.107	0.003	0.560
Dyspnoea	−0.108	−4.962	1.507	0.001	0.570
IPQ-R consequences	−0.085	−2.158	0.866	0.013	0.574

Abbreviations: GP=general practice; HADS=hospital anxiety and depression scale; IPQ-R=illness perception questionnaire; QLQ=quality of life questionnaire.

**Table 4:** Multivariate analysis of all factors from personal and social, disease and treatment, participant-reported co-morbidities, hospital and GP case note reported co-morbidities models and questionnaire models (excluding QLQ-C30 functioning scales)

	**Excluding QLQ-C30 functioning scales (multi-adjusted – *R*^2^=0.538)**				
	**Standardised coefficients *β***	**B**	**s.e.**	***P*-value**	**Cumulative *R*^2^**
*Fixed variables*					
Time since diagnosis	0.107	4.993	1.623	0.002	0.073
Metastatic (or unstaged) disease at diagnosis	−0.079	−4.421	1.863	0.018	0.093
Number of self-reported co-morbidities	−0.072	−1.268	0.590	0.032	0.112
Sex	−0.096	−4.266	1.465	0.004	0.128
					
*Modifiable variables*					
HADS depression subscale	−0.266	−15.440	2.174	<0.001	0.333
Appetite loss	−0.237	−11.458	1.749	<0.001	0.432
Fatigue	−0.189	−10.387	2.014	<0.001	0.485
SDI Q20 difficulty with travel plans	−0.130	−5.778	1.530	<0.001	0.509
IPQ-R consequences	−0.112	−2.874	0.955	0.003	0.520
Dyspnoea	−0.096	−4.450	1.697	0.009	0.530
IPQ-R personal control	0.079	2.157	0.898	0.017	0.535
HADS anxiety subscale	−0.074	−4.066	2.040	0.047	0.538

Abbreviations: GP=general practice; HADS=hospital anxiety and depression scale; IPQ-R=illness perception questionnaire; QLQ=quality of life questionnaire; SDI=social difficulties inventory.
