# Supplementary material for: Modifiable and fixed factors predicting quality of life in people with colorectal cancer
Source: Br J Cancer. 2011 May 10;104(11):1697–703. doi: 10.1038/bjc.2011.155 (PMC3111166; doi:10.1038/bjc.2011.155)
Supplement: Supplementary Tables 1–5 [file bjc2011155x1.doc]

**Supplementary Table 1: Personal and Social Factors**

|  | All PICT data (n=496) | |
| --- | --- | --- |
|  | N | % |
| Age group |  |  |
| Up to age 50 years | 42 | 9 |
| 50-59 years | 96 | 19 |
| 60-69 years | 161 | 33 |
| 70-79 years | 141 | 28 |
| 80 years or over | 56 | 11 |
|  |  |  |
| Mean age (SD) | 66(11.08) |  |
|  |  |  |
| Sex |  |  |
| Male | 273 | 55 |
| Female | 223 | 45 |
|  |  |  |
| Centre |  |  |
| Aberdeen Follow-up | 153 | 32 |
| Aberdeen Newly Diagnosed | 141 | 30 |
| Glasgow Newly Diagnosed | 187 | 39 |
| missing | 15 |  |
|  |  |  |
| Education^a^ |  |  |
| Degree from college/university | 57 | 12 |
| Qualification other than degree from college/university | 94 | 20 |
| O-level/Standard Grade/GCSE/Higher/A-level | 126 | 26 |
| None listed/ or other | 203 | 42 |
| Missing | 16 |  |
| Income |  |  |
| 0 to £10,000 | 106 | 27 |
| £10.000 to £20,000 | 123 | 32 |
| £20,001 to £30,000 | 64 | 16 |
| £30,001 to £40,000 | 41 | 10 |
| £40,001 or more | 56 | 14 |
| Missing | 106 |  |
| Carstairs Quintiles |  |  |
| 1 | 194 | 40 |
| 2 | 117 | 24 |
| 3 | 60 | 12 |
| 4 | 33 | 7 |
| 5 | 87 | 18 |
| Missing | 5 |  |
|  |  |  |
| Urban rural status |  |  |
| Remote | 141 | 29 |
| Accessible | 342 | 71 |
| missing | 13 |  |
|  |  |  |
| Travelling time |  |  |
| < 1hour | 414 | 84 |
| > 1 hour | 82 | 17 |
|  |  |  |
| Home Ownership |  |  |
| Own home | 388 | 79 |
| Rent home | 89 | 18 |
| Other | 15 | 3 |
| Missing | 4 |  |
|  |  |  |
| Living Arrangements |  |  |
| Live alone | 116 | 24 |
| Live with spouse | 321 | 65 |
| Live with family/others | 55 | 11 |
| missing | 4 |  |
|  |  |  |
| Employment status |  |  |
| In paid employment/  self-employed | 151 | 30 |
| Retired | 305 | 62 |
| Other | 40 | 8 |
|  |  |  |
| Smoking status |  |  |
| Never smoker | 188 | 38 |
| Ex smoker | 252 | 51 |
| Current smoker | 54 | 11 |
| Missing | 2 |  |
|  |  |  |

^a^ Where more than one category was ticked the respondent was allocated to the highest qualification category ticked.

**Supplementary Table 2: Disease and Treatment Data**

|  | All PICT data (n=496) | |
| --- | --- | --- |
|  | N | % |
| Time since diagnosis |  |  |
| up to 26 weeks from diagnosis | 317 | 67 |
| ≥48 weeks from diagnosis | 156 | 33 |
| missing | 23 |  |
|  |  |  |
| Site of Cancer |  |  |
| Rectal | 157 | 32 |
| Colon | 269 | 55 |
| Rectosigmoid | 59 | 12 |
| missing | 11 |  |
|  |  |  |
| Recurrence |  |  |
| No | 467 | 94 |
| Yes | 29 | 6 |
|  |  |  |
| Stage at Diagnosis |  |  |
| Complete response to Dukes C | 402 | 81 |
| Metastatic or unstaged | 94 | 19 |
|  |  |  |
| First Contact |  |  |
| Non-emergency or missing | 416 | 84 |
| Emergency | 80 | 16 |
|  |  |  |
| Surgery |  |  |
| Elective surgery | 403 | 81 |
| Emergency surgery | 50 | 10 |
| No surgery | 43 | 9 |
|  |  |  |
| Neo-adjuvant Chemotherapy |  |  |
| None or missing | 400 | 81 |
| Received | 96 | 19 |
|  |  |  |
| Adjuvant Chemotherapy |  |  |
| None or missing | 324 | 65 |
| Received | 172 | 35 |
|  |  |  |
| Palliative Chemotherapy |  |  |
| None or missing | 434 | 88 |
| Received | 62 | 12 |
|  |  |  |
| Neo-adjuvant Radiotherapy |  |  |
| None or missing | 403 | 81 |
| Received | 93 | 19 |
|  |  |  |
| Adjuvant Radiotherapy |  |  |
| None or missing | 485 | 98 |
| Received | 11 | 2 |
|  |  |  |
| Palliative Radiotherapy |  |  |
| None or missing | 475 | 96 |
| Received | 21 | 4 |
|  |  |  |
| STOMA |  |  |
| None or missing | 340 | 69 |
| STOMA | 131 | 26 |
| STOMA reversed | 25 | 5 |
|  |  |  |

**Supplementary Table 3: Patient Reported Co-morbidities**

|  | All PICT data (n=496) | |
| --- | --- | --- |
|  | N | % |
| Number of PRco-morbidities |  |  |
| None | 105 | 21 |
| One | 153 | 31 |
| More than one | 238 | 48 |
|  |  |  |
| Type of PRco-morbidity |  |  |
| No heart disease | 410 | 83 |
| Heart disease | 86 | 17 |
|  |  |  |
| No high blood pressure | 308 | 62 |
| High blood pressure | 188 | 38 |
|  |  |  |
| No diabetes | 440 | 89 |
| Diabetes | 56 | 11 |
|  |  |  |
| No anxiety/depression | 441 | 89 |
| Anxiety/depression | 55 | 11 |
|  |  |  |
| No other cancer | 422 | 85 |
| Cancer (other than bowel) | 74 | 15 |
|  |  |  |
| No osteoarthritis | 419 | 85 |
| Osteoarthritis | 77 | 15 |
|  |  |  |
| Current Pain |  |  |
| No | 216 | 45 |
| Yes | 261 | 55 |
| missing | 19 |  |
|  |  |  |

**Supplementary Table 4: Casenote Co-morbidity Data**

|  | | All PICT data (n=496) | | |
| --- | --- | --- | --- | --- |
|  | | N | | % |
| Mental Health | |  | |  |
| None or missing | | 393 | | 79 |
| Mental Health problem | | 103 | | 21 |
|  | |  | |  |
| Nervous System | |  | |  |
| None or missing | | 491 | | 99 |
| Nervous System problem | | 5 | | 1 |
|  | |  | |  |
| Musculoskeletal | |  | |  |
| None or missing | | 396 | | 80 |
| Musculoskeletal problem | | 100 | | 20 |
|  | |  | |  |
| Cerebrovascular | |  | |  |
| None or missing | | 460 | | 73 |
| Cerebrovascular problem | | 36 | | 7 |
|  | |  | |  |
| Circulatory System | |  | |  |
| None or missing | | 236 | | 48 |
| Circulatory System problem | | 260 | | 52 |
|  | |  | |  |
| Respiratory | |  | |  |
| None or missing | | 421 | | 85 |
| Respiratory problem | | 75 | | 15 |
|  | |  | |  |
| Gastrointestinal | |  | |  |
| None or missing | | 447 | | 90 |
| Gastrointestinal problem | | 49 | | 10 |
|  | |  | |  |
| Endocrine | |  | |  |
| None or missing | | 434 | | 88 |
| Endocrine problem | | 62 | | 12 |
|  | |  | |  |
| Renal | |  | |  |
| None or missing | | 453 | | 91 |
| Renal problem | | 43 | | 9 |
|  | |  | |  |
| Liver | |  | |  |
| None or missing | | 466 | | 94 |
| Liver problem | | 30 | | 6 |
|  | |  | |  |
| Cancer | |  | |  |
| None or missing | | 439 | | 89 |
| Cancer problem | | 57 | | 11 |
|  | |  | |  |
| Hearing Impairment | |  | |  |
| None or missing | | 455 | | 92 |
| Hearing Impairment problem | | 41 | | 8 |
|  | |  | |  |
| Visual Impairment | |  | |  |
| None or missing | | 457 | | 92 |
| Visual Impairment problem | | 39 | | 8 |
|  | |  | |  |
| Number of  HGP Reported Co-morbidities |  | |  | |
| 0 | 98 | | 20 | |
| 1 | 132 | | 27 | |
| 2 or more | 266 | | 53 | |
|  |  | |  | |

**Supplementary Table 5: Questionnaire Data**

|  | |  |  |  |
| --- | --- | --- | --- | --- |
|  | |  | N | % |
| **QLQ-C30** | | |  |  |
| Physical Function Tertiles | | 1 | 161 | 33 |
|  | | 2 | 165 | 33 |
|  | | 3 | 170 | 34 |
|  | |  |  |  |
| Role Function Tertiles | | 1 | 151 | 30 |
|  | | 2 | 182 | 37 |
|  | | 3 | 163 | 33 |
|  | |  |  |  |
| Emotional Function Tertiles | | 1 | 191 | 39 |
|  | | 2 | 149 | 30 |
|  | | 3 | 154 | 31 |
|  | |  |  |  |
| Cognitive Function Tertiles | | 1 | 219 | 44 |
|  | | 2 | 161 | 33 |
|  | | 3 | 115 | 23 |
|  | |  |  |  |
| Social Function Tertiles | | 1 | 166 | 34 |
|  | | 2 | 197 | 40 |
|  | | 3 | 132 | 27 |
|  | |  |  |  |
| Fatigue | | none | 99 | 20 |
|  | | some | 397 | 80 |
|  | |  |  |  |
| Nausea / vomiting | | none | 370 | 75 |
|  | | some | 126 | 25 |
|  | |  |  |  |
| Pain | | none | 242 | 48 |
|  | | some | 254 | 51 |
|  | |  |  |  |
| Dyspnoea | | none | 332 | 67 |
|  | | some | 164 | 33 |
|  | |  |  |  |
| Insomnia | | none | 230 | 47 |
|  | | some | 265 | 54 |
|  | |  |  |  |
| Appetite loss | | none | 347 | 70 |
|  | | some | 149 | 30 |
|  | |  |  |  |
| Constipation | | none | 367 | 74 |
|  | | some | 127 | 26 |
|  | |  |  |  |
| Diarrhoea | | none | 313 | 64 |
|  | | some | 180 | 37 |
|  | |  |  |  |
| Financial problems | | none | 377 | 76 |
|  | | some | 117 | 24 |
|  | |  |  |  |
| **IPQ-R (tertiles)** | | |  |  |
| IPQ Illness identity | | 1 | 196 | 40 |
|  | | 2 | 140 | 28 |
|  | | 3 | 16 | 32 |
|  | |  |  |  |
| IPQ timeline | | 1 | 178 | 36 |
|  | | 2 | 104 | 21 |
|  | | 3 | 209 | 43 |
|  | |  |  |  |
| IPQ timeline cyclical | | 1 | 69 | 14 |
|  | | 2 | 241 | 49 |
|  | | 3 | 179 | 37 |
|  | |  |  |  |
| IPQ consequences | | 1 | 174 | 36 |
|  | | 2 | 127 | 26 |
|  | | 3 | 188 | 38 |
|  | |  |  |  |
| IPQ personal control | | 1 | 164 | 34 |
|  | | 2 | 169 | 35 |
|  | | 3 | 156 | 32 |
|  | |  |  |  |
| IPQ treatment control | | 1 | 122 | 25 |
|  | | 2 | 95 | 19 |
|  | | 3 | 270 | 54 |
|  | |  |  |  |
| IPQ illness coherence | | 1 | 155 | 32 |
|  | | 2 | 73 | 15 |
|  | | 3 | 260 | 53 |
|  | |  |  |  |
| IPQ emotional representations | | 1 | 57 | 12 |
|  | | 2 | 259 | 53 |
|  | | 3 | 173 | 35 |
|  | |  |  |  |
| **SDI** | |  |  |  |
| Q13 difficulties with sexual matters | | none | 356 | 72 |
|  | | some | 140 | 28 |
|  | |  |  |  |
| Q14 difficulty with plans to have a family | | none | 456 | 92 |
|  | | some | 38 | 8 |
|  | |  |  |  |
| Q18 difficulty with where you live | | none | 456 | 92 |
|  | | some | 40 | 8 |
|  | |  |  |  |
| Q20 difficulties with travel | | none | 220 | 44 |
|  | | some | 276 | 56 |
|  | |  |  |  |
|  | |  |  |  |
| Q21 difficulty with any other area of everyday life | | none | 387 | 78 |
|  | | some | 109 | 22 |
|  | |  |  |  |
| SDI summary score | | none | 370 | 78 |
|  | some (score >9) | | 105 | 22 |
|  | |  |  |  |
| **HADS** | |  |  |  |
| Anxiety | | <8 | 396 | 80 |
|  | | ≥8 | 97 | 20 |
|  | |  |  |  |
| Depression | | <8 | 408 | 83 |
|  | | ≥8 | 83 | 17 |
|  | |  |  |  |
